# Pinned Hybrid Glass-Flax Composite Laminates Aged in Salt-Fog Environment: Mechanical Durability

**DOI:** 10.3390/polym12010040

**Published:** 2019-12-26

**Authors:** Luigi Calabrese, Vincenzo Fiore, Paolo Bruzzaniti, Tommaso Scalici, Antonino Valenza

**Affiliations:** 1 Department of Engineering, University of Messina, Contrada Di Dio (Sant’Agata), 98166 Messina, Italy; lcalabrese@unime.it (L.C.); pgbruzzaniti@unime.it (P.B.); 2 Department of Engineering, University of Palermo, Viale delle Scienze, Edificio 6, 90128 Palermo, Italy; antonino.valenza@unipa.it; 3 School of Mechanical and Aerospace Engineering, Queen’s University Belfast, Ashby Building, Stranmillis Road, BT9 5AH Belfast; t.scalici@qub.ac.uk

**Keywords:** bearing, salt fog aging, glass-flax hybrid coposites, pinned joints, failure modes

## Abstract

The aim of the present paper is to study the mechanical performance evolution of pinned hybrid glass-flax composite laminates under environment aging conditions. Hybrid glass-flax fibers/epoxy pinned laminates were exposed to salt-spray fog environmental conditions up to 60 days. With the purpose of assessing the relationship between mechanical performances and failure mechanisms at increasing aging time, single lap joints at varying joint geometry (i.e., hole diameter D and hole distance E from free edge) were characterized after 0 days (i.e., unaged samples), 30 days, and 60 days of salt-fog exposition. Based on this approach, the property–structure relationship of the composite laminates was assessed on these critical environmental conditions. In particular, a reduction of failure strength for long-aging-time-aged samples was observed in the range 20–30% compared to unaged one. Due to the natural fiber degradation in a salt-fog environment, premature catastrophic fractures mode due to shear-out and net-tension were found, related to reduced joint fracture strength. This behavior identifies that this type of joint requires a careful design in order to guarantee an effective mechanical stability of the composite hybrid joint under long-term operating conditions in an aggressive environment.

## 1. Introduction

In the last years, hybrid composite materials were addressed as effective approach to optimize structural design in several industrial fields, such as construction, aerospace or automotive [1,2]. The engineering choice of these materials has shown significant advantages compared to conventional non-hybrid composites [3]. In such a context, the high mechanical properties of synthetic fibers and the environmental compatibility of natural fibers represent an effective and reliable combination to develop hybrid composite materials with marked performances of sustainability and mechanical/structural effectiveness. Several research activates highlighted that the engineering design of natural fiber based composite materials is a potentially suitable key factor to guarantee an effective synergy between mechanical performance and green sustainable material [2,4]. 

Mechanical joint is one of the industrially applied methods to assemble composite structures. Bolted joining is the most common approach to join similar and dissimilar materials thanks to their low cost and easiness in assembling and disassembling. However, structural joints, although often required at the design level, are high stress concentration points that make this region sensitive to damage. Several factors can influence the composite joint strength, such as the preload moment [5], temperature [6], environmental conditions [7], and geometric parameters [8]. The pinned joints with different geometrical parameters highlight different failure modes, and the damage activation phenomena in the composite materials can be foreseen using failure criteria [9].

Several research activities highlighted that the hybridization of lignocellulosic fibers with synthetic counterparts (i.e., glass or carbon fibers) enhances the mechanical properties of composite laminates, favoring the improvement of their tensile, bending, and impact strength [10,11,12,13]. 

Furthermore, the hybridization of natural fibers allows to exalt the durability in critical conditions, preventing the limited durability of the natural materials in moist or wet environments [14,15,16,17]. In fact, it is well known that the performances reduction of GFRP (Glass Fiber Reinforced Plastics) composites in wet condition stabilizes after moisture saturation level [18]. Calabrese et al. [19] showed that the hybridization of lignocellulosic flax fibers with glass ones represents an effective and suitable compromise in terms of environmental impact, mechanical properties, aging resistance, and cost between flax and glass composites, for its use in marine applications. Analogously, Saidane et al. [20] evidenced that the water uptake and diffusion are limited by the addition of glass fiber layers to flax laminates. In particular, the flax-glass hybridization offers a positive contribute in the Young’s modulus and the tensile strength. However, an aspect that needs attention requiring an improvement of knowledge is the evaluation of the mechanical stability in aggressive environments of composite joints based on hybrid natural fibers laminates. Natural fibers exhibit in usual working conditions an acceptable mechanical reliability, configuring these materials as suitable reinforcing material in several applied composites [21,22]. However, due to their relevant hydrophilic nature, they are very sensitive to damp or wet environments, implying a significant performance depletion of the resulting composites, thus addressing premature fracture modes. This makes the mechanical stability and durability design of hybrid joints in aggressive environments very complex. The evaluation of strength variation must be synergistically integrated with the occurred fracture mechanism in the joint [6,23]. The former should be excluded or limited in time, and the latter should preferably be preserved. Instead, there is often the triggering of premature fracture mechanisms, induced by aging, which enhance the limit strength the hybrid joint.

Esendemir and Cabioglu [24] showed that different environmental aging conditions do not modify the failure modes (i.e., net tension, shear-out, bearing, and mixed) for woven glass epoxy pinned composites. Nevertheless, they evidenced that the bearing strength of the joints is noticeable affected by the applied environment conditions.

Karakuzu et al. [25] investigated pinned glass fiber-reinforced composites due to aging up to 12 months of immersion in seawater. Their study evidenced a detrimental effect of this environmental aging condition on failure strength and fracture mechanism of the joint at varying joint geometry. 

In a previous paper, the authors assessed the effect of salt-fog environment on the bearing behavior of pinned flax/epoxy composites [26], showing that salt-fog exposition severely influences the mechanical performances of pinned composite. In particular, a progressive modification of damage mechanism occurred, favoring premature and catastrophic shear out and net tension failure mechanisms despite the preferred bearing one and thus, as a consequence, limiting the effective mechanical durability of the mechanical joint. In such a context, the joint geometrical configuration (i.e., hole diameter, edge distance, etc.) has been a relevant role in the damage activation and propagation. 

At the same time, some promising results were obtained evaluating the durability of glass composite laminates in salt-fog environments [27]. In this case, salt-fog exposition did not modify noticeably the mechanical performances of the pinned flax/epoxy composite. Thanks to the quite good stability in wet environments of glass fibers, only a slight reduction in the performance of the pinned joint was found with no relevant change in fracture mechanisms at increasing aging exposition time. These results encouraged assessment of the hybridization of pinned flax/epoxy composites with glass fibers as an effective approach to enhance the mechanical stability and durability of the joint. Moreover, this would allow improving the knowledge in the hybrid joint design, introducing in this context useful information for a prediction of the component service life based on their real environmental conditions. 

By considering the wide use of composites laminates for marine applications, this issue is extremely interesting to evaluate how the exposition aging time can influence the predominant failure mechanism and, consequently, the structural performance of the hybrid joint. 

In the present paper, pinned glass-flax hybrid reinforced laminates were exposed to salt-fog spray environment up to 60 days with the aim of studying the mechanical performances and failure mechanism modification induced by aging conditions. Different joint geometrical configurations at varying E/D (i.e., hole center to laminate free edge distance over hole diameter) and W/D (i.e., sample width over hole diameter) ratios were also investigated, thus identifying critical values of these parameters that altered the mechanical stability of the joint in a marine environment.

## 2. Materials and Methods 

A composite panel (350 × 350 mm^2^) was produced by using vacuum-assisted resin infusion technique. The was cured at 25 °C for 24 h and post-cured at 50 °C for 8 h. A DEGBA epoxy resin, SX8 EVO (supplied by Mates Italiana, Segrate, Italy) was used as matrix, and 6 layers of 2 × 2 twill weave woven flax fabric with nominal areal weight of 318 g/m^2^ (Lineo, Saint Martin du Tilleul, France) were used as natural fiber reinforcement. In order to improve strength, stiffness, and environmental durability of the composite laminate, 3 layers of plain weave woven glass fabrics with nominal areal weight of 200 g/m^2^ (Mike Compositi, Milano, Italy) were used as external fiber reinforced skins. A full number of 12 reinforced layers (6 internal flax layers and 6 external glass layers) were applied. The stacking sequence of the hybrid composite laminate is (G_3_/F_3_)_s_. 

Prismatic samples with length 150 mm (preferred width 15 mm) were obtained by cutting the panel by using a band saw. Afterward, a single hole was made in each sample by using at first undersized drilling bits and then a mill tool to obtain the hole diameter without edge defects. In order to assess the effect of the pinned joint geometry on mechanical performance and failure mechanisms, hole diameter (D) and its free edge distance (E) were varied in order to obtain samples with large W/D and E/D geometrical ratios.

**Figure 1:**
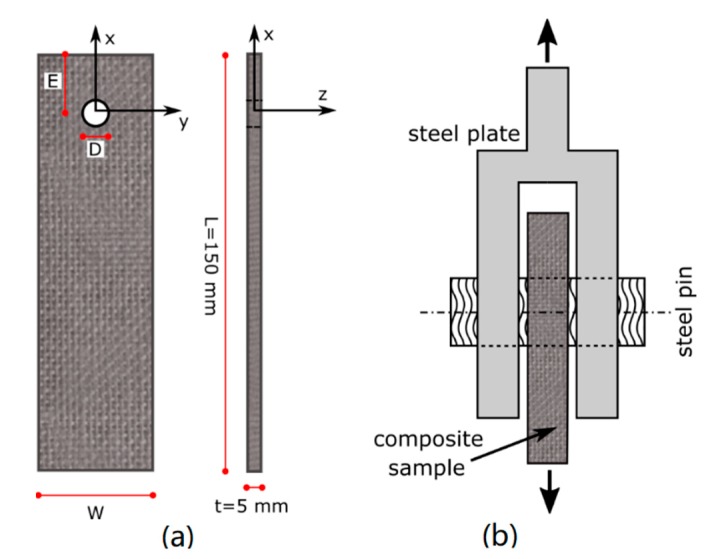
(**a**) Sample geometry and (**b**) bearing test setup.

A double-lap pinned joint was considered for experimental bearing tests, according to ASTM D5961/D standard (procedure A), using a universal testing machine Z250 (Zwick-Roell, Ulm, Germany), equipped with a 250 kN load cell, and setting a displacement rate of 0.5 mm/min. A scheme of the sample geometry and bearing test setup was drawn in Figure 1.

The average fibre and void contents of the laminate, its nominal thickness, the samples geometry, and the bearing test set-up are detailed in our previous paper [28], and it will not reported here for the sake of brevity. 

Aging exposition was carried out by using a DCTC 600 climatic chamber (Angelantoni, Massa Martana (PG), Italy), according to the ASTM B 117 standard. In order to assess the effect of salt-fog exposition on mechanical performances stability of hybrid composite laminates, 30 and 60 aging days were applied on the selected batches. 

All samples are codified as “GFA”, “GFB”, and “GFC” depending on if the hybrid laminates were aged for 0, 30, or 60 days, respectively. Furthermore, this code was coupled with a lot of numbers xx-yy-zz related to the hole diameter (*D*), the edge distance (*E*), and sample width (*W*), respectively. For example, GFB-6-12-15 indicates hybrid laminates exposed to salt-fog for 30 days, having 6 mm hole diameter, 12 mm edge distance, and 15 mm sample width. 

In the present paper, the results and discussion comparison among the three batches was performed by using the bearing stress determined according to following the expression:

(1) σ=P/(D∗s)

where P, D, and s are the applied load, hole diameter, and sample thickness, respectively. Detailed images of failure mechanism were carried out on fractured samples by means of a 3D digital microscope (Hirox KH-8700).

## 3. Results and Discussion

### 3.1. Pinned Joints with 4-mm Hole Diameter

**Figure 2:**
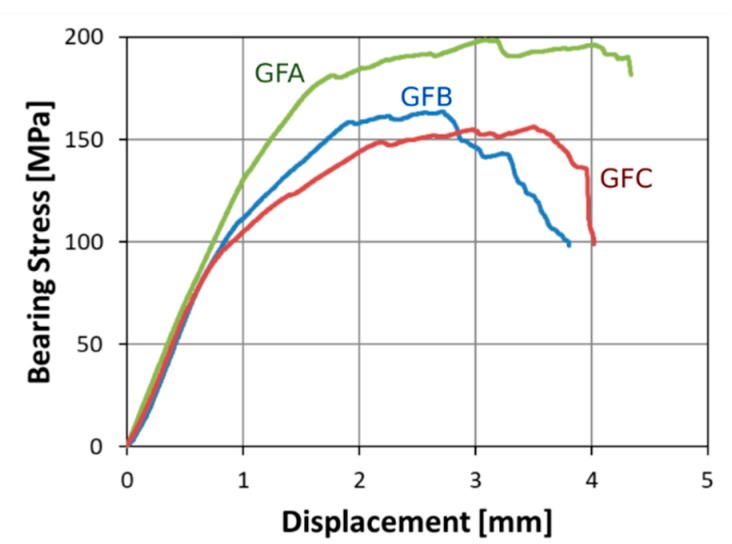
Stress-displacement curves at increasing aging time for pin-loaded laminates (D = 4 mm; E = 12 mm; W = 15 mm).

Figure 2 shows the bearing stress versus displacement trend evolution for hybrid glass-flax laminates having diameter D = 4 mm, width W = 15 mm, and edge distance E = 12 mm (i.e., W/D = 3.75 and E/D = 2.75), as a function of time exposition to salt-fog environment. Three stages can be identified:

- In the beginning phase, all curves are characterized by a linear relationship between the bearing stress and displacement. The slope of this trend could be related to the joint stiffness;
- Afterwards, at increasing displacement, a progressive deviation from the linear trend can be highlighted depending on aging time. This behavior is due to compression collapse of the matrix in correspondence of the composite laminate just behind the small hole/pin contact area;
- Eventually, all specimens tend to reach a plateau zone where the bearing stress is roughly constant, beyond which its failure occurs. All the specimens showed a bearing failure mechanism.

It is worth noting that a gradual modification of the stress-displacement trend can be highlighted at increasing aging time. 

The unaged glass-flax laminate (i.e., GFA sample) evidences effective mechanical performance as confirmed by the high stress level reached before failure. Furthermore, this batch highlights a quite large linearity region for bearing stress values up to about 125 MPa. Then, bearing stress progressively increases at increasing displacement until a stabilization is reached at about 180–190 MPa. A significant stress fluctuation in the bearing stress plateau can be identified due to the compression collapse of the epoxy matrix just behind the pin/hole contact area. A maximum stress at about 200 MPa was reached beyond which the sample failure occurred. 

On the contrary, GFB and GFC samples evidenced a reduction of the maximum bearing stress (i.e., about 18% and 21% lower than unaged sample, respectively) due to salt-fog aging conditions. Moreover, an evident change in plateau region can be observed, i.e., the unaged sample is characterized by a wide plateau region extending in the 1.8–4.2 mm displacement range whereas the stress-displacement curves are characterized in a less extended stabilization region of the stress due to aging condition. This phenomenon is evident already after 30 days of aging exposition (i.e., batch GFB). Flax fibres undergo a gradual absorption of water in salt-fog environment, which leads to a progressive reduction of their mechanical properties. The hydrophilic nature of these fibres also speeds up the water permeation at the fibre-matrix interphase, thus weakening this area and, as a consequence, implying a reduction of both strength and stiffness of the composites due to the limited transfer of stresses [29]. Hence, the residual resistance of the composite laminate decreases, thus promoting the premature compressive collapse of the sample in the pin-hole contact region due to lower stress/deformation limits than unaged hybrid composite laminate. This relevant change of the damage evolution also favours a relevant modification on the occurring failure mechanism. In particular, a transition from full bearing mode to mixed bearing/net tension failure mechanism takes place at increasing exposition time to the salt-fog environment, i.e., 60-day aged samples experienced a clearly mixed net tension and bearing failure mode.

**Figure 3:**
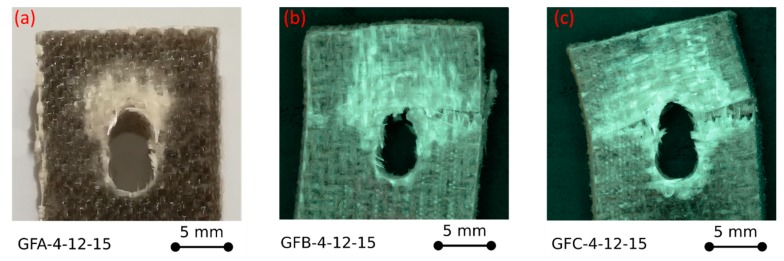
Fracture images of (**a**) GFA-4-12-15, (**b**) GFB-4-12-15, and (**c**) GFC-4-12-15 glass-flax samples.

Further interesting information can be extrapolated by analysing the fracture images of the samples at varying aging time, as reported in Figure 3.

The fracture image of GFA-4-12-15 unaged sample (Figure 3a), fails through bearing mode, as can be evidenced by the large compression collapse area on the pin/hole contact surface area. Furthermore, by analysing the detail of Figure 3a, kink bands that evolve radially from the hole edge can be observed, thus highlighting a progressive extension of the damaged area, defined as failure process zone [28]. This damage phenomenon favours at the same time the triggering of synergistic damage mode, such as delamination, that promotes the mechanical collapse of the pinned laminate joint [30]. In fact, kink bands develop due to plastic micro-buckling phenomena in the compression area and favour large local deformations at the fibre-matrix interface, inducing the triggering of premature secondary failure mechanisms [31]. Therefore, a progressive process of damage accumulation is activated by coupling different damage modes, such as fibre kink bands and shear cracks at the layers interface that evolve to large-scale delamination phenomena, resulting in a reduction in the mechanical stability of the joint [32].

As already stated, the salt-fog exposition induces a significant modification of the failure mechanism. For GFB and GFC samples, a mixed bearing/net-tension fracture was observed (Figure 3b,c). The failure process zone due to bearing is still evident, with a large delaminated area. In these cases, large kink bands toward the free edges of the sample are not evident, indicating that other premature failure mechanisms compete in the damage propagation phenomenon. This can be related to the matrix softening due to environmental aging that limits the local brittle fracture mode such as kink bands and stimulating interfacial dominated fracture mechanisms [33]. The reduced displacement at failure found for GFB and GFC specimens is a consequence of these factors. In particular, the limited plateau region for the specimen GFB (characterized by intermediate exposure times in a salt spray chamber) can be attributed to two competing mechanisms. On the one hand, the onset of a catastrophic fracture mechanism, as net-tension, stimulates the reduction of displacement at failure. On the other hand, the exposure of the sample in a wet environment favors softening phenomena in the composite that reduce the joint stiffness. However, this contribution is mainly relevant for the GFC batch, exposed for 60 days in a salt spray chamber, for which a much larger bearing stress plateau region is detected.

Summarizing, a reduction of about 18% of the bearing stress was evidenced already at 30 days of aging time. Although glass fibres in hydrothermal environments show a quite good mechanical stability, flax fibres, on the other hand, have a limited durability following the reduction of the resistance. These play a key role in triggering damage phenomena that subsequently evolve along the interface surface between dissimilar flax-glass laminae. By observing Figure 3b,c, the appearance of fibre breaking mainly ascribed to the detrimental reduction of the flax tensile strength caused by salt-fog exposition can be evidenced.

**Figure 4:**
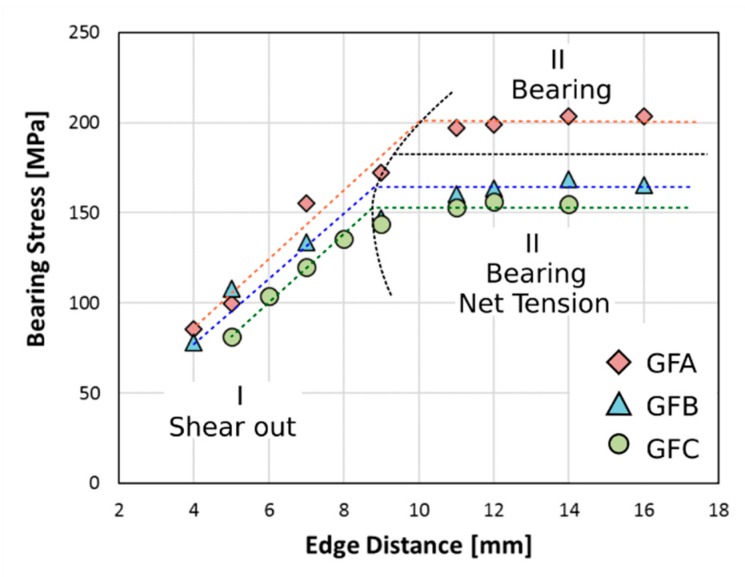
Bearing stress evolution at increasing edge distance E for pin-loaded laminates with hole diameter *D* = 4 mm and width *W* = 15 mm (*W*/*D* = 3.75) for all batches.

Further considerations can be argued by evaluating the maximum bearing stress evolution at increasing edge distance (Figure 4) for samples having constant *D* and *W* (equal to 4 mm and 15 mm, respectively) at varying aging time. The observed experimental trend of all batches (i.e., 0, 20, and 60 aging days) highlights a progressive increase of the maximum bearing stress (σb) at increasing E distance until a critical threshold value, above which its stabilization has been reached, as showed previously [34,35].

For all batches, two linear segments were identified. In particular, F, the pinned composite failure, occurs at very low stress values for small E distance since, the very limited area just behind the pin that suffers the applied stress favours the activation and propagation of premature failure damage by shear out mode (region I). However, as evidenced by the high slope of the first fitting linear segment, the stress is very sensitive to E, i.e., a slight increase in edge distance implies a significant increase in the maximum stress. As already discussed, a threshold E value at about 8–10 mm can be identified. 

For edge distances higher than 10 mm (i.e., E/D > 2.5), a plateau is reached and the bearing stress becomes constant (region II). In this region, the pinned composite joint fails mainly through bearing failure mode [32]. However, after 1 month of aging exposition, glass-flax laminates (i.e., batches B and C) highlighted a combined net-tension and bearing fracture mode with the former dominating on the latter one. Due to salt-fog exposition, hybrid laminates undergo both physical and chemical degradation phenomena that significantly worsen their mechanical behaviour. It is widely known that hydrophilic fibres such as flax ones greatly suffer humid environmental conditions in evidencing gradual reductions in their mechanical properties up to 40% [36]. This sensitivity to damp or wet environments is due to the intrinsic microstructure of the flax fibre, which can be classified as a hierarchical structure reinforced by cellulose micro-fibrils grouped in bundles to form meso-fibrils [37,38]. Thanks to their high elastic modulus (in the range 134 to 160 GPa [39]), these act as reinforcements of the fibers structure. Due to water sorption experienced by the amorphous fraction of cellulose and other polysaccharides such as hemicellulose, a relevant decrease of the mechanical properties of natural fibers occurs [40]. In particular, the cellulose structure is destroyed by the water molecules that lead to a reduction in stiffness, i.e., water acts as a plasticizer increasing the fiber flexibility [41]. Furthermore, the presence of Na+ and Cl- ions in the solution helps to speed up the degradation of epoxy matrix, flax fibers, and their interface by improving the osmotic diffusion of water at the fiber/matrix interface [15]. 

The hydrophilic behavior of the flax fibers enhances the diffusion of water in natural fiber-reinforced laminae. Consequently, this implies an acceleration of the degradation phenomena at the interface between flax and glass reinforced laminae. Furthermore, the water could reach glass fibers, thus partially reducing their mechanical performances due to local dissolution of fiber surface [42]. At the same time. this stimulates a wide activation of softening phenomena of the thermoset matrix [33]. The flax fibers induce the activation of preferential diffusive pathways that enhance the activation and propagation of the conventional degradation phenomena of composites in wet environments. The combination of these phenomena leads to a decrease of the tensile strength due to the water absorption, inducing a significant decrease of net tension failure stress of the aged hybrid composite laminates as early as 1 month of salt-fog exposition. This is confirmed by the reduction of plateau bearing stress for GFB and GFC batches (18% and 24%, respectively) compared to an unaged GFA one. This abrupt stress decrease at high E/D values can be related to the triggering of a premature fracture by net tension that synergistically contributes coupled with bearing on the failure mechanism for this specific joint geometry configuration. 

### 3.2. Pinned Joints with 8-mm Hole Diameter

**Figure 5:**
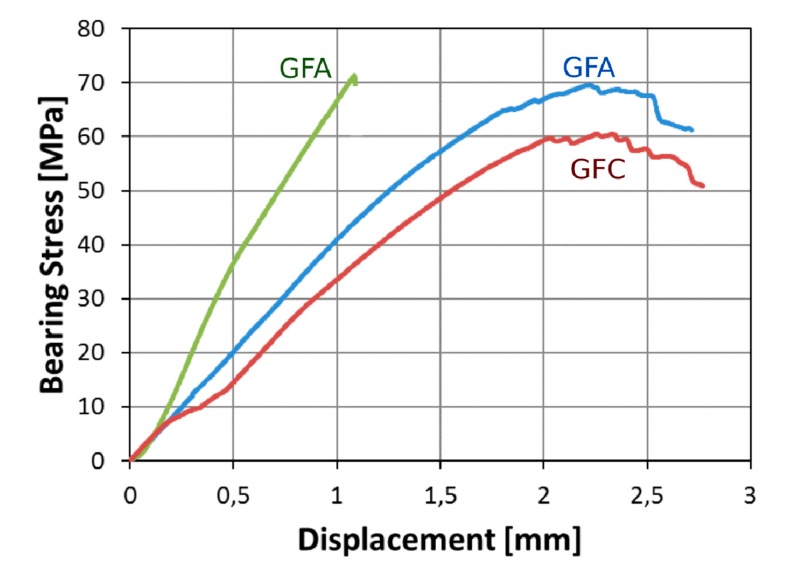
Stress-displacement curves at increasing aging time for pin-loaded laminates (*D* = 8 mm; *E* = 9 mm; and *W* = 15 mm).

The mechanical behavior of glass-flax hybrid laminates can be deeper analyzed by evaluating the stress-displacement trends and fracture mechanisms of pinned composite joints having hole diameter larger than 4 mm. The different geometrical configuration of the joint represents a relevant aspect to be taken into consideration in order to identify the possible causes of triggering and propagation of premature damage on the joint. In particular, Figure 5 shows the evolution of the stress versus displacement curves for pinned joint at increasing aging cycles characterized by hole diameter (*D*) and edge distance (*E*) equal to 8 mm and 9 mm, respectively.

It is worth noting that, for this geometrical configuration, no noticeable decrease of the maximum bearing stress can be observed comparing the three curves although a gradual increase of the displacement at failure can be highlighted at increasing aging time exposition (i.e., 146% and 151% higher than that of unaged one, for batches B and C, respectively). This large deflection for aged samples can be ascribed to two factors. The first is related to a joint stiffness decrease, as evidenced by the reduction of the stress/displacement slope. Moreover, a progressive and not catastrophic damage mechanism occurs at larger deflection values. This favors the stress stabilization, and large deflection is required to induce the final fracture of the aged samples. This behavior, similar to that found for the specimens with a 4-mm hole size, can be attributed to a progressive softening of the matrix and/or matrix-fiber interface. As already stated, this phenomenon is strongly conveyed by the significant sensitivity of flax fibers to water. This is also experimentally confirmed by the reduction of the stress/displacement slope (which may be indirectly related to the joint stiffness) with increasing aging time. After 60 days under salt-fog spray condition, sample GFC-8-9-15 shows a displacement at failure more than double than GFA-8-9-15 unaged sample. 

For a GFA-8-9-15 sample, a catastrophic fracture type was obtained, identifiable by the abrupt and sudden drop of the bearing stress when the stress-displacement trend still has a quite linear relationship. This catastrophic and brittle fracture mechanism can be considered typical for brittle thermoset matrix-based composites [43]. On the other hand, the joint failure takes place prematurely at low stress level for aged samples. Furthermore, the stress-displacement curve does not evidence an abrupt reduction of the stress but a slight and progressive decrease in stress when its maximum value is reached, i.e., this different behavior becomes evident already after 30 days of exposition in the salt-fog environment. As already stated, it can be related to the epoxy matrix softening coupled to the chemical and physical degradation phenomena that can be triggered due to the presence of hydrophilic natural fibers in the hybrid composite laminate (i.e., water diffusion into the composite toward preferential flow pathways, fiber swelling, etc.)

**Figure 6:**
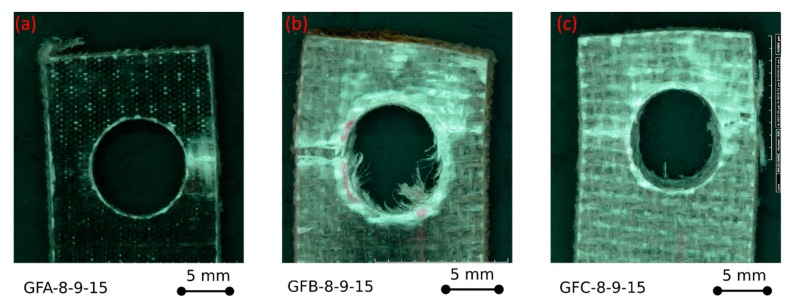
Fracture images of (**a**) GFA-8-9-15, (**b**) GFB-8-9-15, and (**c**) GFC-8-9-15 glass-flax samples.

Figure 6 summarizes the fracture surface for GFA-8-9-15, GFB-8-9-15, and GFC-8-9-15 glass-flax samples).

It is found that, for all the compared samples, a net tension fracture mode mainly occurred. In particular, Figure 6a evidences that an unaged sample fails through a neat fracture surface, typical of tensile failures of fiber reinforced thermoset polymers. No further competing failure mechanisms can be identified. On the other hand, by analyzing Figure 6b,c related to GFB-8-9-15 and GFC-8-9-15 aged samples, respectively, a more complex fracture mechanism can be shown. The net tension fracture area is characterized by local fiber detachments from the matrix coupled to the net-tension crack, local layer detachments, and weakening of the fibers, probably due to the worsening of the fiber-matrix adhesion; to the matrix softening; and to the reduced tensile properties of the natural fibers. At the same time, fracture cracks rivers along the load direction in the area of the composite laminate immediately behind the pinned hole emerge and become more relevant as the exposition time in salt-spray chamber increases. These local damages can be attributed to the onset of joint damaging phenomena due to shear-out mode, which acts synergistically to net-tension one, thus reducing the mechanical strength of the joint. Consequently, this type of fracture although dominated by the fracture for net-tension can be ascribed to an incipient cleavage fracture mode.

**Figure 7:**
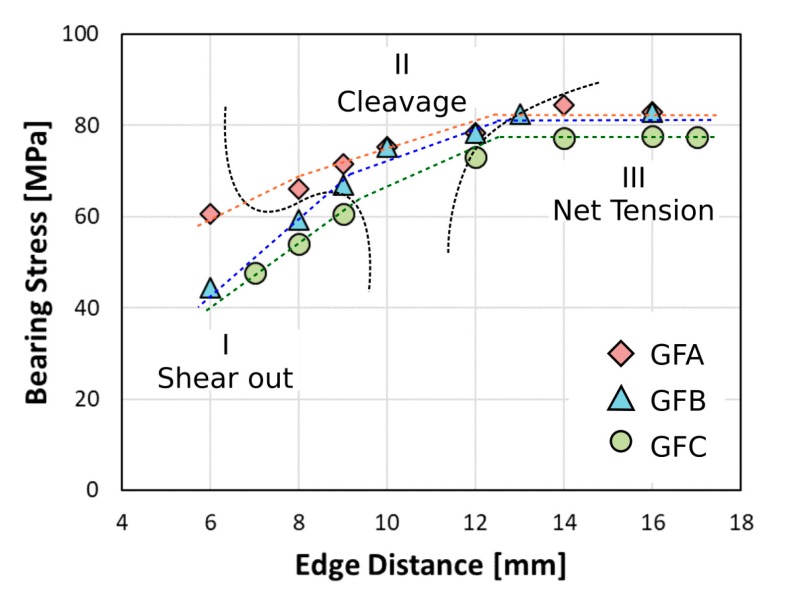
Bearing stress evolution at increasing edge distance E for pin-loaded laminates with hole diameter *D* = 8 mm and width *W* = 15 mm for all batches.

Figure 7 shows the maximum bearing stress evolution at increasing edge distance *E* for pin-loaded laminates with hole diameter *D* = 8 mm and width *W* = 15 mm, for all batches.

For low edge distances (i.e., E < 9 mm), the pinned joints evidenced premature fracture at low stress levels. In particular, for this joint geometrical configuration, shear-out mode is the failure mechanism (region I) due to short free edge distance. The maximum stress increases quite linearly at increasing edge distance up to reaching a threshold value (for *E* = 9). Afterwards, a slight modification of slope trend can be observed, indicating that competing failure phenomena are emerging when pin hole distance from the free edge increases. For all hybrid batches, a transition from shear out to cleavage failure mode occurred (region II). This trend for the GFA batch was maintained up to *E* = 12 mm and then a new failure region due to net tension mode was defined (region III). The curve trend reaches a plateau, indicating that the maximum stress becomes independent from E. It is worth noting that aged samples (i.e., GFB and GFC) evidenced a different failure mechanism at large edge distance: i.e., a mixed mode between net-tension and cleavage. This behavior can be ascribed to a progressive degradation of the composite laminate due to environmental aging that has more markedly affected the laminate shear strength than the tensile strength limit. This is confirmed by the large cleavage failure area evidenced for GFB and GFC samples in Figure 6b,c. Further confirmation of this evidence can be argued by evaluating the relevant slope change in the maximum stress trend in region I (i.e., shear-out region) already at 30 days of exposure in a salt spray chamber (Figure 7). The reduction of the stress value in this region identifies a significant reduction in the shear-out strength of hybrid composite laminates. 

Instead, only a slight modification of maximum stress was shown at large E distance, highlighting a still suitable mechanical stability for this joint configuration, even after long time under salt-fog environment.

The presence of six internal laminae reinforced with hydrophilic flax fibers in the stacking sequence can be considered the main cause of the high water absorption evidenced by hybrid laminates (~6% [19]) during the salt-fog exposition. Furthermore, the water diffusion into the laminate is also influenced by matrix voids and micro cracks due to aging environmental exposure and/or the manufacturing process that speed up the phenomenon [44].

Due to this, a progressive degradation of the hydrophilic flax fibers as well as a weakening of the interfacial adhesion with the surrounding matrix occur during the salt-fog exposition. This justifies the decrement of the specimens’ shear resistance, thus making shear out mode predominant for a wider range of geometrical conditions of aged samples (i.e., GFB and GFC), so remaining competitive with the net tension even at high at large edge distance. 

### 3.3. Evolution of Failure Mechanisms at Varying Joint Geometry

**Figure 8:**
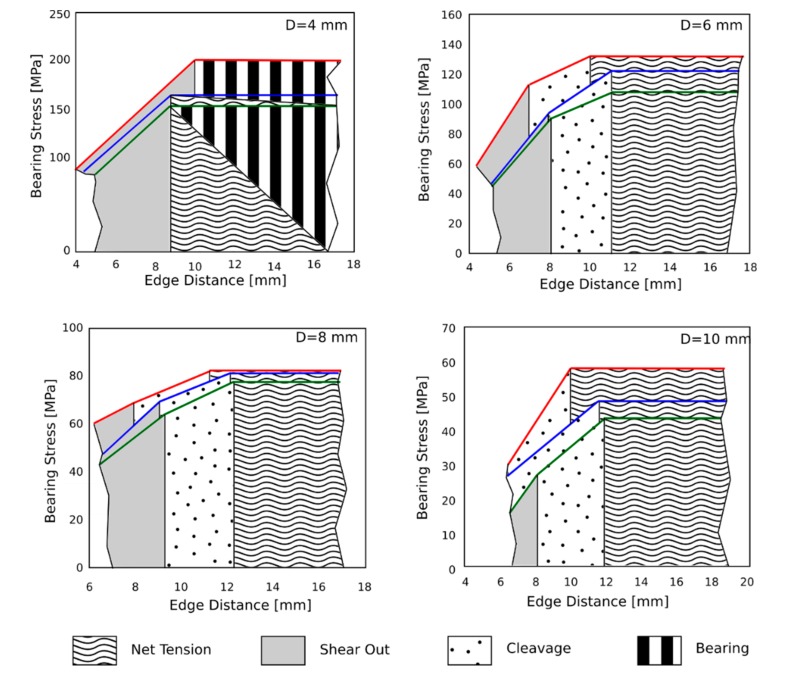
Scheme of failure mechanisms evolution in bearing stress vs. edge distance E plot for all laminates.

In order to better discriminate the damage mechanisms that compete at varying joint geometry, in Figure 8, different topological failure plots obtained evaluating the maximum stress at varying the geometric parameters E (edge distance) and D (hole diameter) were schemed.

Some consideration can be addressed based on this schematization:

- *D* = 4 mm: For a small hole diameter, the fracture mechanism evolves from shear-out (i.e., low E values) to bearing mode (i.e., large E values). Nevertheless, a premature fracture caused by net-tension fracture mode was highlighted for large edge distance value due to environmental aging. For the aged samples, a mixed bearing/net tension fracture was identified. The maximum stress decreases at increasing aging time. This behavior is more sensitive for joint geometry having large edge distance and, therefore, large E/D ratio.
- *D* = 6–8 mm: For intermediate hole diameters, a dual failure damage mechanism occurs. A transition from shear-out to net-tension failure mechanism takes place at increasing edge distance. For this sample geometry, the cleavage mode is relevant, especially for aged specimens. This behavior can be related to the reduced tensile and shear strengths of the aged flax fibers that influences the triggering of combined failure mechanisms. GFB and GFC batches, compared to the unaged GFA one, are characterized by a clearly lower stress in the shear-out region (mainly for samples with hole diameter equal to 6 mm). This significant stress reduction is related to the specific joint geometry since the difference between hole diameter and sample width is still relevant, thus limiting net-tension fracture mechanism only at high E values. The low stress evidenced in the shear-out failure region could be related to a relevant reduction of the interfacial adhesion at the fiber/matrix interface that favors shear cracks activation and propagation in the pin/hole contact area. This stimulates a premature fracture by shear-out, thus preventing a mixed failure mechanism for cleavage.
- *D* = 10 mm: For large hole diameters, net-tension is the main failure mechanism for *E* > 10–12 mm for aged and unaged samples. This experimental evidence indicates that all samples, regardless of the applied aging time, exhibit quite similar fracture transitions. Nevertheless, a relevant discrepancy on the mechanical strength was observed. In particular, GFB and GFC samples showed a reduction of maximum stress of about 20% and 30% compared to unaged one for *E* > 12 mm, respectively. This trend can be attributed to the reduced tensile strength in the hydrophilic aged flax fibers. This degradation phenomenon is less relevant at low E/D ratio, where the shear-out mode is the main failure mechanisms.

These results show that the hybridization procedure of flax fibers using glass fibers allows to obtain composite materials with an acceptable aging stability up to 60 days of exposure to salt spray environment. In particular, a reduction of the maximum stress of about 20–30% can be identified due to aging. The presence of not hydrophilic glass fibers, placed externally in the stacking sequence, made it possible to limit the phenomenon of water absorption [15]. Hybridization on flax composites improves the durability of the composite. This allowed to obtain an acceptable mechanical stability [26]. These results are in agreement with Reference [19], where the hybridization of lignocellulosic fibers (i.e., flax) with synthetic ones (i.e., glass) was possibly considered if accurately designed and suitably applied for marine applications based on its compromise in terms of environmental impact, mechanical properties, aging resistance, and cost between flax and glass composites.

However, the triggering of premature fracture mechanisms at low stress levels, such as shear-out and net-tension, are a symptom of the partial interaction of the hybrid composite with wet environments. Thus, these environmental conditions are at potentially critical state at long exposure times.

Summarizing, due to the natural fiber degradation in salt-fog environment, at varying *D* and *E* dimensions, premature catastrophic fractures mode due to shear-out and net-tension were found, related to reduced joint fracture strength. This behavior identifies that this type of joint requires a careful mechanical durability design in an aggressive environment in order to guarantee an effective mechanical stability of the composite hybrid joint under long-term operating conditions.

## 4. Conclusions

In the present paper, the effect of salt-fog environment on the mechanical behavior of pinned hybrid glass-flax/epoxy composites was evaluated. Samples at varying geometrical joint configuration were exposed to a salt-fog spray test up to 60 aging days, according to ASTM B 117 standard.

The experimental results highlighted that salt-fog environmental conditions significantly modify the mechanical performances and failure mechanism of pinned hybrid composites. For low hole diameter (i.e., *D* = 4 mm), a reduction of the bearing strength of about 18–21% was observed due to environmental aging, i.e., from 198.7 to 156.1 MPa for GFA-4-12-15 and GFC-4-12-15 samples, respectively. Furthermore, it was evidenced a noticeable modification of damage mode at increasing the aging exposition time. In particular, a reduction of the bearing phenomenon thus favoring premature and catastrophic mechanisms such as shear out (i.e., for low E values) and net tension (i.e., for high E values) was highlighted and, as a consequence, limited the effective mechanical durability of the mechanical joint.

This greater sensitivity to net-tension and shear-out fracture evolution is exalted also for joints having larger hole dimensions. In particular, for hole size D equal to 10 mm, it was observed a bearing strength reduction of about 30% when the fracture dominant mechanism was both shear out (i.e., for E values lower than 8 mm) and net-tension (i.e., for E values higher than 12 mm), respectively. 

In conclusion, the main goal of the present paper consists in the evaluation of the effect of glass-flax hybridization on the durability of pinned composite laminates, i.e., a deeper knowledge of the stability of hybrid composite components in salt-fog environments was achieved. In particular, the experimental approach may help the design phase with the aim of optimizing the performance of the composite structures in terms of mechanical stability and durability. Future studies will be addressed to improve this knowledge by investigating the mechanical stability of these composite materials in other critical environmental aging conditions complementary to salt spray setup (e.g., wet/dry cycles).

## References

[1] Pervaiz M., Panthapulakkal S., KC B., Sain M., Tjong J. (2016). Emerging Trends in Automotive Lightweighting through Novel Composite Materials. Mater. Sci. Appl..

[2] Fragassa C. (2017). Marine applications of natural fibre-reinforced composites: A manufacturing case study. Advances in Applications of Industrial Biomaterials.

[3] Pošvář Z., Růžička M., Kulíšek V., Mareš T., Doubrava K., Uher O. (2016). Design of Composite Hybrid Structures “on Request”. Mater. Today Proc..

[4] Mastura M.T., Sapuan S.M., Mansor M.R., Nuraini A.A. (2017). Environmentally conscious hybrid bio-composite material selection for automotive anti-roll bar. Int. J. Adv. Manuf. Technol..

[5] Zou P., Cheng H. Effect of interference-fit percentage and preload on the mechanical behaviour of single-shear lap composite joint. Proceedings of the IOP Conference Series: Materials Science and Engineering; Institute of Physics Publishing.

[6] Calabrese L., Fiore V., Scalici T., Bruzzaniti P., Valenza A. (2019). Failure maps to assess bearing performances of glass composite laminates. Polym. Compos..

[7] Fiore V., Calabrese L., Proverbio E., Passari R., Valenza A. (2017). Salt spray fog ageing of hybrid composite/metal rivet joints for automotive applications. Compos. Part B Eng..

[8] Lv J., Xiao Y., Xue Y. (2018). Time–temperature-dependent response and analysis of preload relaxation in bolted composite joints. J. Reinf. Plast. Compos..

[9] Valenza A., Fiore V., Calabrese L. (2010). Three-Point Flexural Behaviour of GFRP Sandwich Composites: A Failure Map. Adv. Compos. Mater..

[10] Zhang Y., Li Y., Ma H., Yu T. (2013). Tensile and interfacial properties of unidirectional flax/glass fiber reinforced hybrid composites. Compos. Sci. Technol..

[11] Mandal S., Alam S. (2012). Dynamic mechanical analysis and morphological studies of glass/bamboo fiber reinforced unsaturated polyester resin-based hybrid composites. J. Appl. Polym. Sci..

[12] Fiore V., Valenza A., Di Bella G. (2012). Mechanical behavior of carbon/flax hybrid composites for structural applications. J. Compos. Mater..

[13] Ramesh M., Palanikumar K., Reddy K.H. (2016). Influence of fiber orientation and fiber content on properties of sisal-jute-glass fiber-reinforced polyester composites. J. Appl. Polym. Sci..

[14] Pan Y., Zhong Z. (2015). The effect of hybridization on moisture absorption and mechanical degradation of natural fiber composites: An analytical approach. Compos. Sci. Technol..

[15] Fiore V., Calabrese L., Di Bella G., Scalici T., Galtieri G., Valenza A., Proverbio E. (2016). Effects of aging in salt spray conditions on flax and flax/basalt reinforced composites: Wettability and dynamic mechanical properties. Compos. Part B Eng..

[16] Retegi A., Arbelaiz A., Alvarez P., Llano-Ponte R., Labidi J., Mondragon I. (2006). Effects of hygrothermal ageing on mechanical properties of flax pulps and their polypropylene matrix composites. J. Appl. Polym. Sci..

[17] Fiore V., Scalici T., Sarasini F., Tirilló J., Calabrese L. (2017). Salt-fog spray aging of jute-basalt reinforced hybrid structures: Flexural and low velocity impact response. Compos. Part B Eng..

[18] Garcia-Espinel J.D., Castro-Fresno D., Parbole Gayo P., Ballester-Muñoz F. (2015). Effects of sea water environment on glass fiber reinforced plastic materials used for marine civil engineering constructions. Mater. Des..

[19] Calabrese L., Fiore V., Scalici T., Valenza A. (2019). Experimental assessment of the improved properties during aging of flax/glass hybrid composite laminates for marine applications. J. Appl. Polym. Sci..

[20] Saidane E.H., Scida D., Assarar M., Sabhi H., Ayad R. (2016). Hybridisation effect on diffusion kinetic and tensile mechanical behaviour of epoxy based flax-glass composites. Compos. Part A Appl. Sci. Manuf..

[21] Peças P., Carvalho H., Salman H., Leite M. (2018). Natural Fibre Composites and Their Applications: A Review. J. Compos. Sci..

[22] Li X., Tabil L.G., Panigrahi S. (2007). Chemical treatments of natural fiber for use in natural fiber-reinforced composites: A review. J. Polym. Environ..

[23] Machado J.J.M., Gamarra P.M.R., Marques E.A.S., da Silva L.F.M. (2018). Improvement in impact strength of composite joints for the automotive industry. Compos. Part B Eng..

[24] Esendemir Ü., Ayşe A.M. (2013). Investigating bearing strength of pin-loaded composite plates in different environmental conditions. J. Reinf. Plast. Compos..

[25] Karakuzu R., Kanlioǧlu H., Deniz M.E. (2018). Effect of seawater on pin-loaded laminated composites. Mater. Test..

[26] Fiore V., Calabrese L., Scalici T., Valenza A. (2020). Evolution of the bearing failure map of pinned flax composite laminates aged in marine environment. Compos. Part B Eng..

[27] Calabrese L., Fiore V., Bruzzaniti P.G., Scalici T., Valenza A. (2019). An Aging Evaluation of the Bearing Performances of Glass Fiber Composite Laminate in Salt Spray Fog Environment. Fibers.

[28] Fiore V., Calabrese L., Scalici T., Bruzzaniti P., Valenza A. (2018). Bearing strength and failure behavior of pinned hybrid glass-flax composite laminates. Polym. Test..

[29] Fiore V., Scalici T., Calabrese L., Valenza A., Proverbio E. (2016). Effect of external basalt layers on durability behaviour of flax reinforced composites. Compos. Part B Eng..

[30] Sola C., Castanié B., Michel L., Lachaud F., Delabie A., Mermoz E. (2016). On the role of kinking in the bearing failure of composite laminates. Compos. Struct..

[31] Opelt C.V., Cândido G.M., Rezende M.C. (2018). Compressive failure of fiber reinforced polymer composites–A fractographic study of the compression failure modes. Mater. Today Commun..

[32] Fiore V., Calabrese L., Scalici T., Bruzzaniti P., Valenza A. (2018). Experimental design of the bearing performances of flax fiber reinforced epoxy composites by a failure map. Compos. Part B Eng..

[33] Malmstein M., Chambers A.R., Blake J.I.R. (2013). Hygrothermal ageing of plant oil based marine composites. Compos. Struct..

[34] Abd-El-Naby S.F.M., Hollaway L. (1993). The experimental behaviour of bolted joints in pultruded glass/ polyester material. Part 1: Single-bolt joints. Composites.

[35] Turvey G. Failure of single-lap single-bolt tension joints in pultruded glass fibre reinforced plate. Proceedings of the 6th International Conference on Composites in Construction Engineering (CICE).

[36] Assarar M., Scida D., El Mahi A., Poilâne C., Ayad R. (2011). Influence of water ageing on mechanical properties and damage events of two reinforced composite materials: Flax-fibres and glass-fibres. Mater. Des..

[37] Baley C., Le Duigou A., Bourmaud A., Davies P. (2012). Influence of drying on the mechanical behaviour of flax fibres and their unidirectional composites. Compos. Part A Appl. Sci. Manuf..

[38] Bos H.L., Van Den Oever M.J.A., Peters O.C.J.J. (2002). Tensile and compressive properties of flax fibres for natural fibre reinforced composites. J. Mater. Sci..

[39] Mohanty A.K., Misra M., Hinrichsen G. (2000). Biofibres, biodegradable polymers and biocomposites: An overview. Macromol. Mater. Eng..

[40] Akil H.M., Cheng L.W., Mohd Ishak Z.A., Abu Bakar A., Abd Rahman M.A. (2009). Water absorption study on pultruded jute fibre reinforced unsaturated polyester composites. Compos. Sci. Technol..

[41] Dhakal H.N., Zhang Z.Y., Richardson M.O.W. (2007). Effect of water absorption on the mechanical properties of hemp fibre reinforced unsaturated polyester composites. Compos. Sci. Technol..

[42] Wei B., Cao H., Song S. (2011). Degradation of basalt fibre and glass fibre/epoxy resin composites in seawater. Corros. Sci..

[43] Ouarhim W., Zari N., Bouhfid R., Qaiss A. (2019). el kacem Mechanical performance of natural fibers–based thermosetting composites. Mechanical and Physical Testing of Biocomposites, Fibre-Reinforced Composites and Hybrid Composites.

[44] Fiore V., Scalici T., Badagliacco D., Enea D., Alaimo G., Valenza A. (2017). Aging resistance of bio-epoxy jute-basalt hybrid composites as novel multilayer structures for cladding. Compos. Struct..

